# An Unusual Accidental Diagnosis of a Myocardial Infarction

**Published:** 2012-10-11

**Authors:** Angelica Fundaliotis, Fabio Pastore, Gianluigi D’Alessandro, Rosalba Romano, Marino Paolo

**Affiliations:** 1 Division of Cardiology, Azienda Ospedaliera-Universitaria “Maggiore della Carità”, Eastern Piemonte University, Novara, Italy.; 2 Division of Cardiology, Maria Cecilia Hospital, Cotignola, Italy.; 3 Department of Medicine, Salerno University, Italy

A 69-year-old man came to our observation after a cerebral vascular stroke. Six years before our observation, the patient had been subjected to a pericardiocentesis for an episode of cardiac tamponade. The procedure had been performed without any complication. After the procedure, the patient had been asyntomatic until September 2010, when he had been hospitalized in our hospital for a cerebral ischemia in the left premotor frontal area, followed by a successful recovering.

Two months later, he had a recurrent ischemic stroke. The electrocardiogram showed a slight progression of the R waves in the precordial leads and Q waves in the inferior leads. Therefore additional diagnostic procedures were performed, including a transthoracic echocardiogram (Figure 1, left panel) that revealed the presence of an inferior large dyskinetic area with a large stratified thrombus [1].

We additionally performed a cardiac MRI (Figure 1, right panel) that confirmed the presence of a large pseudoaneurysm (7.3 x 6.3 x 5.1 cm) of the left ventricular (LV) medium-basal inferior wall, with a large thrombus affixed to the wall with a maximum thickness of 1.5 cm. Cardiac MRI showed a fixed perfusion defect and a marked signal hyperintensity (late enhancement) of the LV posterior wall [2].

The coronary angiogram showed a long critical stenosis in the distal segment of the right coronary artery, without any significant stenosis of the left coronary artery. After the diagnostic phase, the patient underwent cardiac surgery removing the pseudoaneurysm. Surgery showed a rupture of LV inferior wall, with a superimposed thrombus (residual gap of 4x3 cm). The pseudoaneurism was removed and the LV wall was repaired with a Dacron patch and the layer of biological glue [3].

This case further supports the importance of performing an echocardiogram to exclude any potential cardiac cause in case of recurrent cerebral strokes.

## Figures and Tables

**Figure 1:**
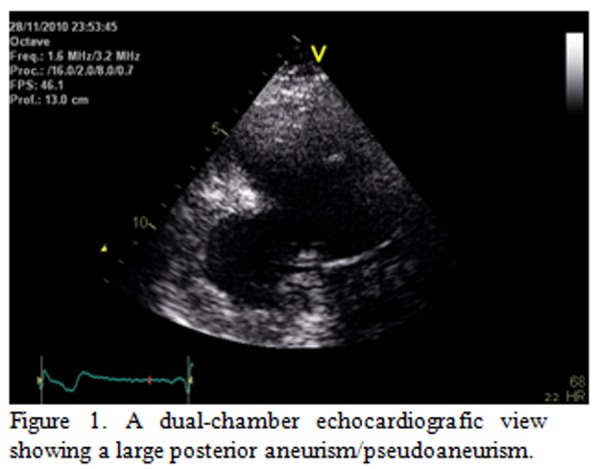
A dual-chamber echocardiografic view showing a large posterior aneurism/pseudoaneurism.

**Figure 2:**
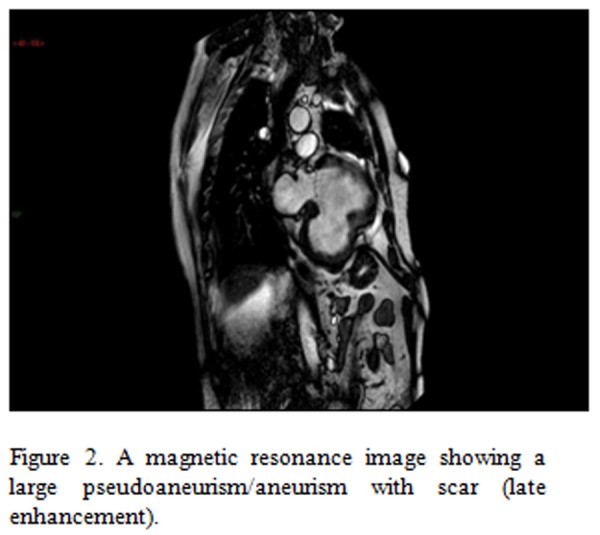
A magnetic resonance image showing a large pseudoaneurism/aneurism with scar (late enhancement).

**Figure 3:**
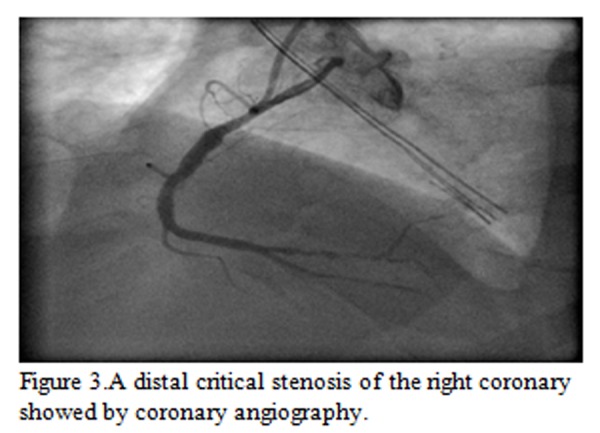
A distal critical stenosis of the right coronary showed by coronary angiography.
